# The rising young scientist stars in China

**DOI:** 10.1007/s13238-016-0347-5

**Published:** 2016-11-29

**Authors:** Hao Cheng

**Affiliations:** Beijing Institutes of Life Science, Chinese Academy of Sciences, Beijing, 100101 China

The Chinese government has devoted a great amount of effort to strengthen its scientific capacity, as an important national strategic aim. Increased funding support, improving scientific structure and administration combined with rapid economic growth has seen great achievements in science and technology by Chinese scientists over the past decade. China has now become an outstanding country with advanced developments in major disciplines, especially in biology.

As of 2016, more than 40 manuscripts have been published in top-tier research journals, such as *Cell*, *Nature*, and *Science* from Chinese Research Groups. These outstanding discoveries are contributed by not only well-known senior world-class biologists, such as Feng Shao, George F. Gao, Yigong Shi and Qi Zhou, but also some young scientists including Maojun Yang, Nieng Yan, Wei Xie and Zhenfeng Liu. In this article, three young Chinese scientific stars will be introduced with their research work and impact in their relevant fields.

## Junjiu Huang

**Figure 1:**
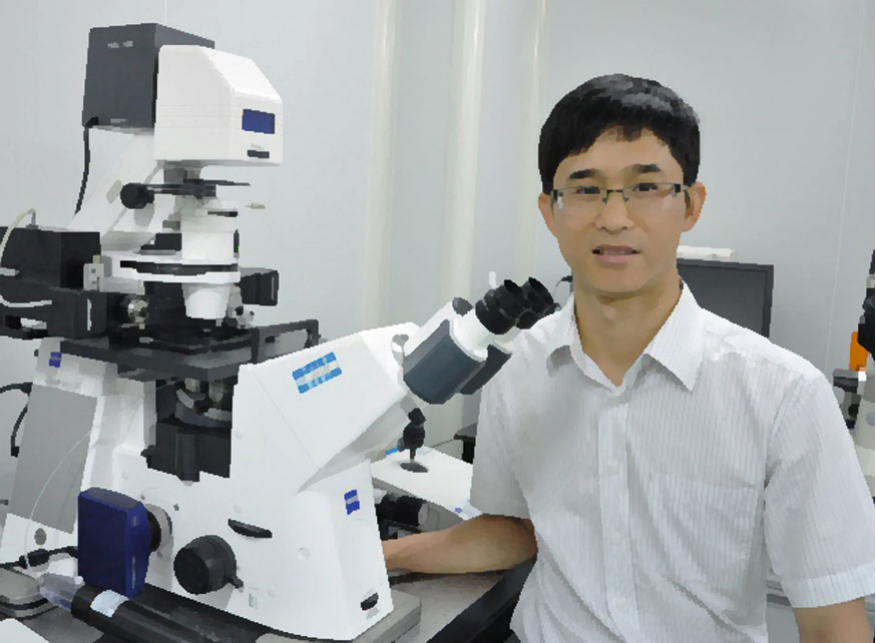
Dr. Junjiu Huang

Prof. Junjiu Huang (Fig. 1) from Sun Yat-sen University, Guangzhou, and his team accomplished the first genomic editing of human embryos, which is recognized as a great breakthrough on gene editing. Confronting with unfair challenges and bias from some vindictive peer scientists, he published this manuscript on *Protein & Cell* in May 2015 determinedly and sparked a high-profile debate on editing the genomes of human embryos (Liang et al., 2015).

In this study, Prof. Huang used tripronuclear (3PN) zygotes (“non-viable” embryos) to investigate the specificity and fidelity of the CRISPR/Cas9 system. Similar to cultured human cells, most of the double strand breaks (DSBs) generated by Cas9 in 3PN zygotes were also repaired through non-homologous end joining (NHEJ). He successfully corrected the gene responsible for the blood disorder β-thalassaemia (a potentially fatal blood disorder), though some unexpected mutations were generated. His name is highlighted as one of “ten people who mattered this year”, in one of the leading scientific journals, *Nature*, in 2015. “It can show genetic problems related to cancer or diabetes, and can be used to study gene function in embryonic development.” “Our paper was just basic research, which told people the risk of gene editing.” Prof. Huang replied to the controversy with the scientific viewpoint. A recent article in *Nature* demonstrated the editing of the mutations in the β-globin (HBB) gene of haematopoietic stem cells (HSCs) also by the CRISPR/Cas9 system, which represented a strategy for the next generation of β-thalassaemia therapies based on gene editing, proving the importance of Prof. Huang’s experiments (Dever et al., 2016).

Furthermore, Prof. Huang has made important contributions on the mechanism of cell senescence and regeneration, and the mechanism of early implantation embryo development in mammals. He published several influential research articles in journals *Molecular Cell*, *Stem Cells*, *Aging Cell*, *Fertility and Sterility*, and overviewed “telomere regulation in pluripotent stem cells” for *Protein & Cell* in 2014 (Huang et al., 2014). He was selected in the Young Talents of Guangdong Province for “thousand, hundred and ten excellent” Special Program, Pearl River Nova of Guangzhou, and also won the “Stem Cell Young Investigator Award” of Chinese Society for Stem Cell Biology. As a modest and easygoing molecular biologist, Prof. Huang shows his determination on pursuit for truth by exploring the unknown areas, regardless of opposition.

## Maojun Yang

**Figure 2:**
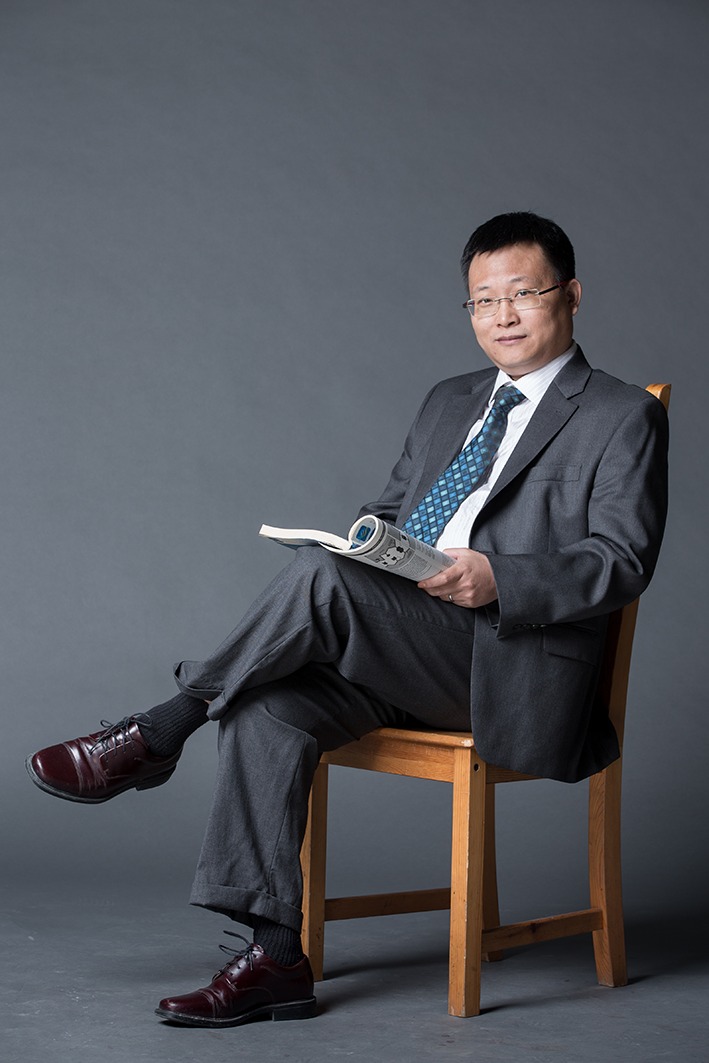
Dr. Maojun Yang

In September 2016, a manuscript entitled “The Architecture of the Mammalian Respirasome” was published in *Nature* by Prof. Maojun Yang (Fig. 2) from the School of Life Sciences, Tsinghua University (Gu et al., 2016). This article provided the highest resolution to date the cryo-EM structure of the mammalian mitochondria respirasome, a large and important protein molecular machine involving in the mammalian respiratory system. This was based on his previous findings on the structure of type-II mitochondria complex I, which was published in *Nature* in 2012 (Feng et al., 2012). Prof. Yang confidently stated, “More detailed results about how the respirasome works will be demonstrated by our lab and we hope these results will be published soon”. His results have provided insight into the organization of mammalian respiratory chain, revealed completely new mechanisms of proton pumping and electron transfer. As the reviewers wrote “This achievement can therefore be considered as a real breakthrough in the field of bioenergetics because it allows for the first time to get insight in how the small mobile electron carriers do their job between the components.”, and “I believe the manuscript is a landmark study and should be of great significance”.

Prof. Yang received his training as a crystallographer from 2001 to 2003, under the supervision of Prof. Zihe Rao in Tsinghua University. During his PhD and post-doctoral training, he became an expert on protein crystal structure, mainly focusing on the spindle checkpoint system by using the X-Ray method. He has authored more than 10 research articles in high impact scientific journals including *Cell*, *PLoS Biology*, *Molecular Cell*, *PNAS*, *Nature Structural & Molecular Biology*, etc. As a new technique, cryo-EM technique has exhibited numerous advantages compared to X-Ray on protein structure research. Prof. Yang therefore decided to learn the single particle cryo-EM technique. Due to his strong determination, he became an expert on cryo-EM technique in less than 2 years, successfully solving complicated membrane protein structures of the mammalian Piezo1 (Ge et al., 2015) and mitochondria respirasome with the single particle cryo-EM technique.

It is well-known that Prof. Yang is an ambitious workaholic, staying up entire nights without eating and sleeping in order to solve a protein structure when finds a significant clue. He has also set a high standard for research. He told his students that the projects from his lab must be to answer meaningful scientific problems, and that they must face the challenges and conquer them, regardless of difficulty. Prof. Yang has given much support to *Protein & Cell*, publishing several important structure papers (Chai et al., 2013; Wang et al., 2013; Zhang et al., 2015; Zhuo et al., 2014). He wrote two review articles entitled “Amazing structure of respirasome: unveiling the secrets of cell respiration” to summarize the research milestones on the mitochondria respiratory chain in detail (Guo et al., 2016) and “When MAGE meets RING: insights into biological functions of MAGE proteins ” to describe the molecular mechanism of how the cancer testis antigen functions as a cofactor in protein ubiquitination systems (Feng et al., 2011).

## Yanhui Xu

**Figure 3:**
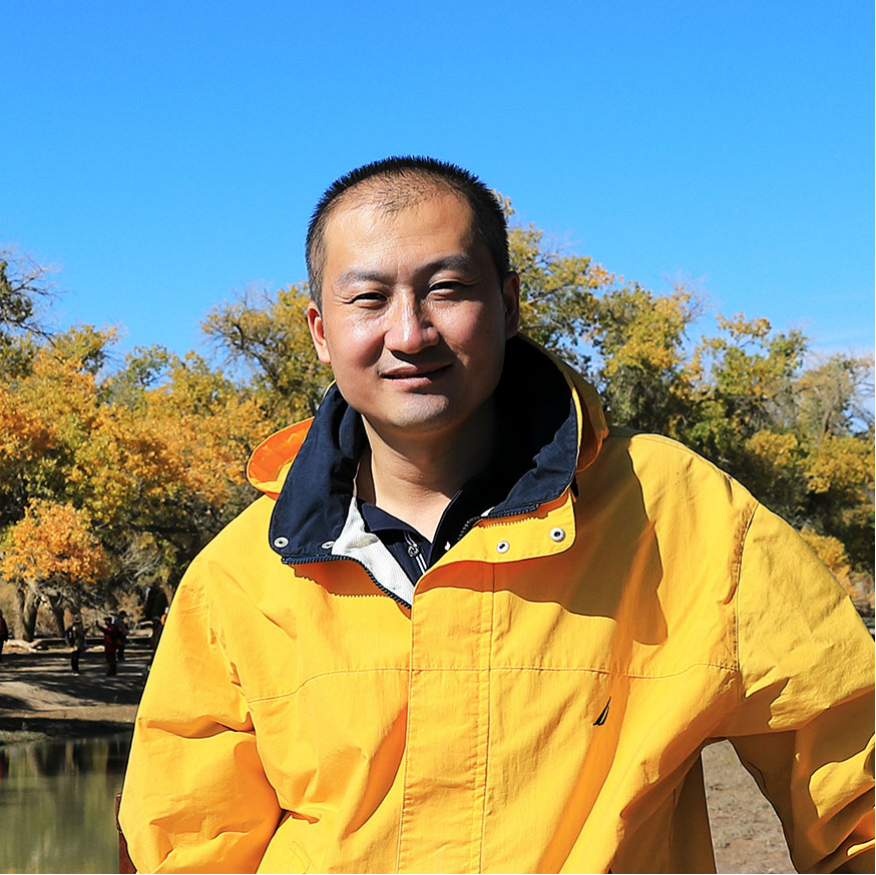
Dr. Yanhui Xu

Prof. Yanhui Xu (Fig. 3) from Institutes of Biomedical Sciences, Fudan University, published his landmark work, in the form of two manuscripts to *Nature* in 2015. The studies revealed underlying mechanisms for catalysis, substrate recognition, and regulation of enzymatic activity of epigenetic regulators by providing structural data of DNMT3A-DNMT3L and DNMT3A-DNMT3L-H3 complex, as well as DNMT1 in complex with USP7, which received extensive attention from peer scientists worldwide (Hu et al., 2015; Guo et al., 2015). With the research interest in exploring the mechanism of oncogenesis and development, young passionate scientist Dr. Xu was appointed as a professor and established his lab at Fudan University in 2008. In 2013, Prof. Xu determined the complex crystal structure of TET2 bound to methylated DNA, displaying the molecular mechanism for TET-mediated 5mC oxidation and DNA demethylation (Hu et al., 2013). This outstanding work was accepted by *Cell*, with positive comments by reviewers, and was recommended by *Faculty of 1000*, *Nature China* and previewed in *Cell*. Furthermore, he presented crystal structure data for some important elements of DNA methylation and cancer metabolism in *Protein & Cell*, such as USP_7_, KDM_5_B and PKM_2_ (Cheng et al., 2015; Wang et al., 2015; Zhang et al., 2014).

Looking back on Prof. Xu’s education experience, it is obvious why he can achieve such important discoveries. He published over 10 manuscripts in 5 years during his graduate studies in Tsinghua University, under the supervision of Prof. Zihe Rao. Working as a post-doctoral fellow in Princeton University, USA, he authored two publications in *Cell*, one publication in *Molecular Cell*, and one publication in *Nature Structural & Molecular Biology* within 4 years. Talented and hardworking, Prof. Xu was honored numerous awards, including the Shanghai Pujiang Scholar, Shanghai Shuguang Scholar, New Century Excellent Talents Awards, National Science Fund for Distinguished Young Scholars, Changjiang Scholars Program—Distinguished Professor, China Outstanding Youth Award for Science and Technology, etc. More tough scientific problems on molecular mechanism of cancer will be solved by Prof. Xu’ s lab in the future.

However, there are some unknown young scientists who simply want to take the short-cut to become scientific stars, without putting in the hard work and dedication required. By doing this, these young scientists risk their career with unauthentic articles, containing disputed and unrepeatable data.

On May 2, 2016, Dr. Chunyu Han from Hebei University of Science and Technology reported his new gene-editing technology called NgAgo, in the journal *Nature Biotechnology* (Gao and Wang 2016). Although his research initially received numerous plaudits and recognition from both domestic and international scientists, his research has currently the subject of academic fraud, since other scientists are unable to repeat Dr. Han’s results. Furthermore, the controversy is becoming more intense as domestic and international scientists are calling on Dr. Han to provide his original data (Gao et al., 2016).

To realize an innovation-driven development environment in China, the government encourages and supports ambitious young scientists to develop their own talent and wisdom on basic scientific research and transformation of science and technology. However, scientific integrity needs to be strictly and faithfully maintained, and all scientific activities should be supervised.

Since 2001, the Chinese government has already designed national plans for promoting basic scientific research to support upgrading traditional industries, propelling hi-tech research, strengthening basic researches, deepening reform in the system of science and technology, and building a state system of innovation. According to Innovation-Driven Development Strategy Outlines of China, these national policies will definitely benefit research development and attract more young promising scientists to achieve their scientific goals in China.
